# Green Extraction of Plant Materials Using Supercritical CO_2_: Insights into Methods, Analysis, and Bioactivity

**DOI:** 10.3390/plants13162295

**Published:** 2024-08-18

**Authors:** Metin Yıldırım, Mehmet Erşatır, Samet Poyraz, Madina Amangeldinova, Nataliya O. Kudrina, Nina V. Terletskaya

**Affiliations:** 1 Department of Biochemistry, Faculty of Pharmacy, Harran University, Sanliurfa 63050, Türkiye; 2 Department of Chemistry, Faculty of Art and Science, Cukurova University, Adana 01330, Türkiye; mersatir@cu.edu.tr; 3 Independent Researcher, Nevşehir 50040, Türkiye; s.poyraz88@gmail.com; 4 Department of Biodiversity and Biological Resources, Faculty of Biology and Biotechnology, Al-Farabi Kazakh National University, Al-Farabi Av., 71, Almaty 050040, Kazakhstan; madu.ma@mail.ru (M.A.); kudrina_nat@mail.ru (N.O.K.); teni02@mail.ru (N.V.T.); 5 Institute of Genetic and Physiology, Al-Farabi Av., 93, Almaty 050040, Kazakhstan

**Keywords:** supercritical fluid, extraction, supercritical carbon dioxide, medicinal plants, anticancer activity, green chemistry

## Abstract

In recent years, the supercritical CO_2_ extraction method has gained attention due to its use of environmentally friendly, non-toxic solvents, ability to operate at lower temperatures that do not cause the degradation of bioactive compounds, and capacity for rapid extraction. This method is particularly notable for isolating bioactive compounds from plants. The extracts obtained have shown superior properties due to their activity against diseases such as cancer, which is one of the leading causes of death worldwide. The aim of this study is to provide an in-depth understanding of the supercritical CO_2_ extraction method, as well as to discuss its advantages and disadvantages. Furthermore, the study includes specific data on various plant materials, detailing the following parameters: plant name and region, bioactive compounds or compound classes, extraction temperature (°C), pressure (bar), time (minutes), co-solvent used, and flow rate. Additionally, this study covers extensive research on the isolation of bioactive compounds and the efficacy of the obtained extracts against cancer.

## 1. Introduction

Population growth and the increase in factories are leading to escalating climate crises and environmental problems. Solvents play a crucial role in the chemical industry and chemical reactions, and despite their frequent use, they are known to impact the environment, cost, safety, and health. Solvents are involved in numerous essential processes, including cleaning, extraction, chemical synthesis, and the synthesis of pharmaceuticals [1]. Solvents such as acetone, ethanol, methanol, dichloromethane, hexane, and ether are commonly used in the extraction processes of medicinal plants, including maceration, Soxhlet, ultrasound-assisted, and microwave-assisted extraction, due to their high efficiency [2]. However, solvents can harm the eyes, skin, lungs, kidneys, and other organs [1].

It is essential to avoid the use of agents that harm the environment. Therefore, the development and implementation of environmentally sensitive technologies in daily life are imperative. One of the main goals of green chemistry is to prevent or minimize the environmental damage caused by chemicals used in chemical processes [3,4]. The supercritical carbon dioxide (ScCO_2_) extraction method has garnered interest from scientists in recent years as an extraction method that uses carbon dioxide as a supercritical solvent [5,6]. This method can prevent or minimize the use of organic solvents, reduce storage problems associated with organic solvents, and consequently, minimize environmental issues [7].

Between 2000 and 2024, there have been over 7800 research studies related to “supercritical CO_2_ fluid extraction” in the SCOPUS database (as of 21 June 2024). Therefore, the details of this promising technology should be well understood and further developed. In recent years, the emergence of new diseases, along with the side effects and inadequacies of traditional drugs in treating existing diseases, has led to an increasing interest in natural methods that have been used since ancient times. Therefore, the activities of extracts obtained from medicinal plants used in folkloric medicine should be thoroughly investigated by scientists [2].

Instead of using environmentally harmful solvents to obtain these extracts, the preference and development of sustainable methods such as ScCO_2_ extraction are necessary for a more livable world. In recent years, studies on extracts prepared with this method in preclinical research for cancer, the second deadliest disease in the world, are more frequently encountered in the literature. The aim of this study is to comprehensively investigate the supercritical extraction method and compile preclinical studies in the literature up to 2024 on plant extracts obtained with this method against cancer.

This study differs from other reviews by offering comprehensive theoretical information on the ScCO_2_ extraction method. Additionally, it explores the anticancer effects of the plant extracts obtained through this method, along with the characterization methods employed. Furthermore, it delves into the synergistic effects observed when these extracts are combined with existing anticancer drugs.

### 1.1. Supercritical Fluid

**Figure 1:**
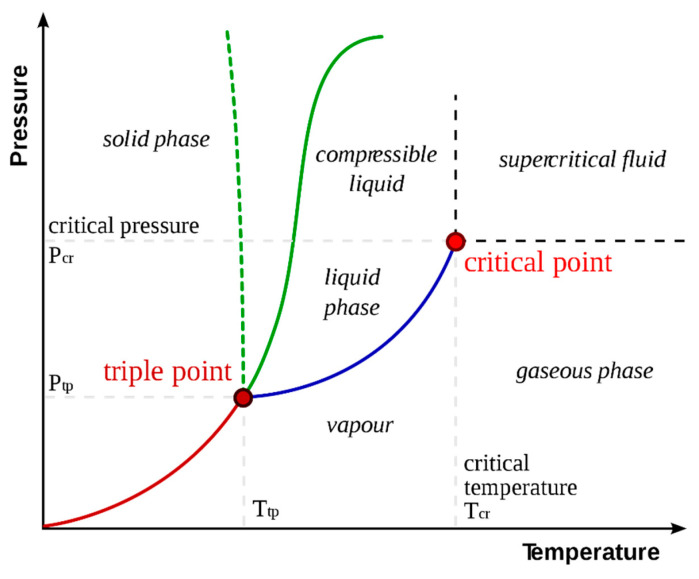
Phase diagram [1].

In the pressure–temperature phase diagram of a substance, as the gas–liquid equilibrium curve is moved forward, its temperature and pressure increase, while the density of the liquid decreases due to thermal expansion; as the pressure increases, the density of the gas begins to increase. Gradually, the densities of the two phases approach each other, and the differences between gas and liquid disappear and the end of the curve reaches a critical point. At this point, matter can now be called “fluid”. Thus, when the temperature of the substance is increased above its critical temperature (Tc) and its pressure above its critical pressure (Pc), a new region, different from the solid, liquid, and gas phases, emerges and the fluid in this region is defined as “supercritical fluid (SF)” (Figure 1) [8]. It was first explained by Hannay and Hogart, at the Royal Society seminars (London) in 1879, that a solid dissolves in a gas at high pressure, and that the solid collapses when the pressure is reduced [9]. The physicochemical properties of SF are between those of liquids and those of gases. This feature makes SF a more effective solvent.

The critical point is the temperature and pressure at which the liquid and gas phase cannot be distinguished. At this point, all physical properties of liquid and vapor are the same. The lines separating the liquid, vapor and solid, phases are phase boundaries. These lines show the change in phase, and in these lines, the two phases are in balance. An SF is a substance that is above the critical temperature and below the pressure. At the triple point, matter exists as a combination of three phases. When we increase the temperature and pressure along the liquid/gas line, the difference between these two phases eventually disappears and they become identical and single in appearance.

#### Basic Physical Properties of SFs

The densities of SFs are similar to those of liquids, and their viscosity and diffusivity are similar to those of gases. As the density increases, the solvation power of SFs increases and they can dissolve more substances than gases (Table 1) [10]. With the increase in diffusivity and decrease in viscosity, the SF can easily diffuse like gases in the pores of the solid structure and its dissolving power increases. The physical properties of SFs vary in a wide range depending on temperature and pressure. However, these properties are generally somewhere between the values of liquids and gases [11].

Intensity: When the temperature is kept constant, it increases strongly with increasing pressure, whereas, when the pressure is kept constant, it decreases with increasing temperature. An SF has the density of a liquid (between 0.1 and 1.0 g/mL) and its characteristic resolving power. Thus, the resolving power can be varied by temperature and/or pressure adjustment. Diffusion is higher in SFs than it is in the liquid state. Viscosity is lower than it is in the liquid state. These physical properties of the SFs enable mass transfer to be rapid [12]. The physical properties of some fluids are given in Table 2.

### 1.2. CO_2_ as a SF

The critical pressure value (Pc) of ScCO_2_ is 72.9 atm and the critical temperature value (Tc) is 31 °C. These temperature and pressure values belong to the critical point (CP). From this point on, carbon dioxide is neither a liquid nor a gas. At the triple point (TP) of carbon dioxide, a mixture of solid, liquid, and gas exists in equilibrium. In the region between the triple point and the critical point, the substance is a liquid; in the part below the vapor pressure line, it is a gas; and in the part above the sublimation and condensation line, it is a solid [14,15].

The physicochemical properties (density and viscosity) of ScCO_2_ at different temperatures and pressures are given in Table 3. The density and viscosity of ScCO_2_ increases with increasing pressure at a constant temperature; However, at a constant pressure, it is seen that the density and viscosity values decrease with increasing temperature, and small changes in temperature and pressure cause significant changes in the physicochemical properties of ScCO_2_ [16].

Another physical property that affects the solubility of substances in SFs is the dielectric constant (ε), which optimizes solubility and reaction conditions in various chemical processes. The dielectric constant refers to a material’s capacity for polarization under an electric field, and this value directly affects the material’s solvent properties and behavior in various chemical reactions [17]. As seen in Table 4, the dielectric constant is inversely proportional to temperature and pressure, and in some cases, it behaves parallel to density [18].

The solvent power of ScCO_2_ can be defined as a function of its density. The solubility power of ScCO_2_ can be controlled by controlling the temperature and pressure of the system [19]. The density of ScCO_2_ changes markedly with pressure and temperature in the region close to the critical temperature and pressure values. While the resolving power of CO_2_ is quite low at ambient conditions (the density of CO_2_ is 2.0 kg·m^−3^), it is quite high at the critical point (the density of CO_2_ is 470 kg·m^−3^) [19]. Small changes in the temperature and pressure of SFs cause the density of the fluid to change. This property affects the resolving power of SFs.

#### 1.2.1. Advantages of ScCO_2_

ScCO_2_ is a released gas that can be purchased at a low price. It can replace other organic solvents used in industry, thereby preventing the release of toxic substances into the environment. This method also contributes to the reduction of the carbon footprint. As a non-toxic solvent, it finds applications in various fields such as pharmaceuticals [20], the food industry [21], the environment [22], and crude oil extraction [21]. The ability to conduct processes at low temperatures is a significant advantage, as it helps preserve synthetic active pharmaceutical ingredients or natural active compounds found in plants that are susceptible to thermal degradation. The CO_2_ used in the process can be collected and reused with the aid of specialized equipment. This process is rapid and selective, allowing for the isolation of desired compounds at appropriate temperatures and pressures [9,11,12,13,14,16,23,24,25].

#### 1.2.2. Disadvantages of ScCO_2_

Despite being a cost-effective method, ScCO_2_ requires a high initial investment for the necessary equipment. Additionally, one of the most crucial aspects is the training provided to operators. Due to the complexity of the process, inadequately trained operators can cause potential malfunctions in the equipment. Furthermore, this method operates at high pressures, posing safety risks due to potential equipment failures and gas leaks. One of the primary limitations is the solubility issues caused by the apolar nature of CO_2_. To overcome this problem, many studies utilize co-solvents [21]. The optimization of process variables such as pressure and temperature is required, leading to additional time and costs. [9,23].

In the last decade, the use of ScCO_2_ instead of organic solvents has gained significant momentum [26,27]. In addition to its low critical point, a dissolving power comparable to that of liquids, and diffusion properties similar to those of gases, ScCO_2_ is considered environmentally friendly. From an environmental standpoint, research indicates that ScCO_2_ will be beneficial in synthesis, extraction, and textile dyeing in the future. The quantity of research papers related to ScCO_2_, including various subjects, methods, and procedures, is progressively growing on a daily basis. Studies on ScCO_2_ are generally focused on pharmacology and drugs, polymers, polymer additives, textile dyes, natural products (tobacco and coffee), foods (milk, etc.), surfactants and cleaning agents, aerogels, foams, cosmetic products, fossil fuels, explosives, rocket fuels, oils, lipids, and various catalysts [15,24,26,27]. The techniques and methods developed in studies on these issues can be listed as particle design, micronization, recrystallization, various reactions and syntheses, hydrogenation, hydroformylation, extraction, fractionation, and chromatography. Among these research topics related to ScCO_2_, catalyst synthesis and solubility in ScCO_2_ interest us more. In fact, it is noticeable that the increased use of ScCO_2_ instead of organic solvents has been evident in many synthesis studies carried out in recent years. While ScCO_2_ has solvent properties like those of liquids and diffusion properties like those of gases, providing suitable reaction conditions, this fluid can be easily removed from the reaction environment by reducing the pressure, making these studies noteworthy. As ScCO_2_ begins to replace commonly used organic solvents, it also eliminates the effect of organic solvents on selectivity.

Physical properties such as density, diffusion coefficient, viscosity, and dissolution force are determined by the pressure and temperature values in the supercritical region. These properties can be greatly changed by small adjustments in pressure and temperature. Unlike liquid solvents, the viscosity and diffusion coefficients of SFs are close to those of gases, and they have a density similar to that of liquids. SFs have higher mass transfer properties compared to liquid solvents due to their low viscosity and high diffusion coefficients [24]. In general, with these advantageous properties of fluids, the low surface tension of ScCO_2_ facilitates the recycling of synthesized substances with a simple expansion, allowing them to be easily separated from the SFs. The fact that the viscosity of SFs is 10 times lower than that of liquids and the diffusion speed of solute molecules in this environment is 10 times greater are considered advantages for SFs [13]. SFs possess a notable characteristic due to their high density (0.2–0.5 g/cm^3^), which is their exceptional capacity to dissolve substantial non-volatile compounds. For example, CO_2_ at supercritical conditions easily dissolves n-alkanes with 5–30 carbon atoms, di-n-alkyl phthalates with alkyl groups with 4–16 carbon atoms, and polycyclic aromatic hydrocarbons consisting of polyrings. Many industrial processes also rely on the high solubility of organic substances in ScCO_2_. For example, this solvent is used to extract caffeine from coffee to obtain decaffeinated coffee. Additionally, to produce low-nicotine cigarettes, nicotine in tobacco is extracted in the same way with ScCO_2_ [13]. Another feature of SFs is that the analytes dissolved in these fluids can be easily recovered from their solutions by equilibrating them with the atmosphere at a relatively low temperature. For example, an analyte dissolved in ScCO_2_ can be recovered by reducing the pressure on the solution and evaporating the solvent under normal laboratory conditions. This property is crucial for analytes sensitive to temperature changes [13]. Not only that, but several projects have already made use of the ScCO_2_ reaction medium for organic syntheses due to its inertness, non-toxicity, non-flammability, low cost, easy availability, and environmental compatibility [13].

### 1.3. Parameters Affecting Efficiency in Supercritical Fluid Extraction (SFE)

The main factors affecting the extraction efficiency in studies conducted with SFE are the temperature, pressure, flow rate, and modifiers used. Apart from these parameters, sample grinding, the sample size, the drying agents, etc., used also have an effect. Although other parameters are influential, they are less discussed among studies. The following sections provide more details on these four important parameters.

#### 1.3.1. Temperature

Changing the temperature affects the extraction efficiency because it changes the solubility of the solute. Because the solubility and extraction efficiency are directly proportional, the solvent density and solute vapor pressure (volatility) change with the temperature. These factors have a direct relationship with solute solubility. The vapor pressure of the solute increases with the temperature, resulting in increased solubility, whereas increasing the temperature reduces the solvent concentration and, subsequently, the solubility and extraction efficiency decrease [28,29,30]. There is no clear reason for this situation. Differences in the content, size, and structure of various plants may be the main reason for the different results in the functionality of heat. The yield of many compounds varies depending on the temperature. For instance, in the extraction of parboiled rice bran oil, different temperatures (40, 60, and 80 °C) were used, and the highest yield was obtained at 40 °C [31]. In another study, lycopene was extracted from tomatoes using ScCO_2_ at temperatures ranging from 313 to 373 K. According to the obtained data, it was found that higher amounts of lycopene were obtained as the temperature increased [32].

#### 1.3.2. Pressure

The most effective parameter in extractions with SFs is pressure. Increasing the pressure increases the solvent density and the solvation power of the SF, resulting in increased extraction efficiency. High pressures increase the density of ScCO_2_, thereby reducing mass transfer resistance and facilitating the isolation of both apolar and polar compounds. However, high-pressure conditions do not guarantee the isolation of all apolar compounds. Additionally, careful consideration must be given when increasing the pressure settings during the process, as high pressure can affect the stability of certain active compounds. Moreover, high pressure also increases operational costs [33].

#### 1.3.3. Flow Rate

The flow rate, defined as the amount of CO_2_ that passes through the plant matrix in a given time period, is a crucial factor that directly impacts the efficiency and effectiveness of extraction. The flow rate controls the length of time ScCO_2_ stays in contact with plant material; this period has a major effect on dissolution and transport kinetics. In general, high flow rates make the extraction process faster and shorter, but they also raise the risk of components not being dissolved or transported thoroughly enough. Reduced flow rates let the supercritical fluid interact with the plant material for a longer period of time, therefore facilitating more efficient component dissolving; but this can extend the extraction time and raise the running costs. The ideal flow rate achieves the highest possible efficiency in transporting and transferring dissolved components, while also maintaining a balance between energy consumption and equipment performance. Precise regulation of the flow rate is crucial for enhancing the effectiveness of the extraction process and guaranteeing the highest possible yield of the targeted components [34,35,36,37].

#### 1.3.4. Modifier

CO_2_ has a low polarity and is less effective at removing polar bioactive material embedded in the plant cell wall. Therefore, for the extraction of plant extracts enriched in polar compounds, such as many phenolic compounds, polar auxiliary solvents such as water, ethanol, or methanol, called modifiers, are used, which are added in small amounts along with the SF [26,29,38].

Modifiers, for example, are cosolvents that can effectively change the behavior of supercritical fluid ScCO_2_ and move it into a subcritical state. This shift can present a number of obstacles, one of which is the difficulty of removing the modifiers from the fluid after processing. The use of modifiers can impact the critical temperature and pressure of CO_2_, thereby compromising its beneficial characteristics such as its low viscosity, high dispersibility, and exceptional solvent qualities.

Furthermore, the separation of modifiers from ScCO_2_ following processing may provide challenges, and this alteration may impair the effectiveness of procedures that depend on the special qualities of supercritical CO_2_, such as enhanced mass transfer and solubility.

Modifiers can establish robust affinities with CO_2_ or solutes, rendering their total elimination challenging without supplementary procedures. This results in heightened processing expenses and necessitates the utilization of more energy-intensive or less eco-friendly techniques to ensure efficient separation.

Hence, although modifiers can be advantageous in adjusting the characteristics of ScCO_2_ for certain purposes, their utilization must be meticulously controlled to prevent compromising the advantages of employing ScCO_2_ and to guarantee the efficiency and environmental sustainability of the separation process [39].

#### 1.3.5. Grinding Time and Particle Size

The herbal mixture can have a positive effect on the quality and yield of extracts. Many parameters, such as particle size, shape, surface area, porosity, etc., have an impact on SFE results. Grinding the plants and producing small particles has a positive effect on the extraction efficiency [26,29,40]. In fact, smaller particles provide more surface/volume for supercharged fluids, resulting in a decrease in internal mass transfer resistance and, subsequently, an increase in the yield of the extracts. However, increasing the grinding time may have a negative impact on the extraction process. If the grinding time is long, the temperature of the samples and the device may increase, which may affect the process. This can be explained by the fact that some volatile compounds move away from the extract and, therefore, the extraction efficiency decreases.

### 1.4. Extraction of Biologically Active Substances from Plants with ScCO_2_

Plants are the most abundant natural entities in nature that contain pharmacologically important components [41,42]. Natural products are used as a source for cosmetics, dyes, pharmaceuticals, food, and traditional medicine [27,28,38,43,44]. Various chemicals derived from plants are used to prevent or fight various diseases such as cancer, cardiovascular disorder, antifungal or antibacterial disease, neurological disorder, and diabetes [26,29,37,44,45,46,47,48]. Plants are a rich source of secondary products such as alkaloids, glycosides, and tannins [49]. The extraction of these substances from plants is a very important process in drug discovery and new drug development. The importance of the extraction process, which involves separating the active element from the crude drug with a suitable solvent, for medical sciences cannot be ignored. The extraction journey, which started with traditional methods used for years, has accelerated with the ultramodern use of SFs. As we expect better quality drugs, there is an increase in studies conducted with better quality extraction techniques. The aim of the researchers is to obtain valuable compounds in improved yields with low solvent residue. Traditional extraction methods are manual processes such as boiling, filtration, maceration, hydrodistillation, and soxhlet. Such extraction methods are generally simple and practical. However, they have disadvantages such as being laborious, using solvents that require additional steps for removal, and using heat, in many techniques, thus leading to the degradation of heat-sensitive molecules. Most traditional methods are not completely effective, require large amounts of organic solvents, and are expensive due to the need to dispose of this organic waste, which, in itself, can pose an environmental risk.

The use of SFE is a breakthrough for the extraction of biologically active compounds. In the last two decades, this method has transitioned from laboratory scale to industrial scale. The emergence of green chemistry has emerged with the aim of reducing energy consumption, performing experimental processes with high yields in a short time, and replacing traditional solvents with less environmentally harmful alternatives. Some of the green and modern technologies used for phytoextraction are SFE, ultrasound-assisted extraction, mechanical pressing, microwave-assisted extraction, and instant softening control (DIC). Among these methods, SFE is a method of separating or extracting chemical compounds from the matrix at a critical temperature and pressure as the extraction solvent. Carbon dioxide is the most commonly used solvent in SFE due to its important advantages such as easy availability, easily accessible critical temperature and pressure (31 °C and 72.9 bar), being non-toxic and inert, and easy separation from the matrix. To date, important processes such as the decaffeination of coffee with ScCO_2_ extraction, hops extraction, catalyst regeneration, and the extraction of organic wastes from water and soil have been carried out [9,24,50]. In this review, studies carried out in recent years with ScCO_2_ extraction as a modern technique for the extraction of biologically active compounds from plants are included. Extraction examples using ScCO_2_ in recent years are given in Table 5.

#### Preparation of Plant Material for ScCO_2_ Extraction

The time of collection of the plant to be used in the extraction process from its natural environment affects the content of the extract to be obtained. Features such as the season determined for the collection, the time of the day, the specified location, and whether the collection is performed on a rainy day or a dry day are important because the chemical composition of the plant changes. The plant to be used for the extraction process can also be purchased from the market or herbalists. After the plant is collected, whichever part is to be extracted (flower, fruit, stem, root, bark, seed, etc.) is carefully separated from other plant materials, and then the plant is dried naturally or by applying heat in oven-like systems. Steps such as grinding and drying can significantly affect the phytochemical content of plant material. To prevent the enzymes in the plant from functioning improperly and to halt plant metabolism, it is necessary to remove the water present in the plant. The drying temperature should be appropriate to prevent microorganism growth and to preserve the chemical content of the plant. Drying can be done under either artificial or natural conditions.

Natural drying involves leaving the plant material in open air or semi-open rooms where there is no direct sunlight. In artificial conditions, the drying process can be controlled by adjusting the temperature and pressure. To preserve volatile components and prevent the degradation of compounds, the drying temperature should be carefully selected. Techniques such as freeze-drying, vacuum chambers, ovens, tunnel dryers, and shaft dryers are commonly used in laboratories for this purpose.

Before supercritical fluid extraction, the plant’s moisture content should be low (<10%), and the particle size of the material should be as small as possible. It is important to employ techniques that do not damage the plant’s volatile components and phytochemical content while reducing these parameters. As the moisture content increases, the extraction yield decreases. Additionally, the smaller the particle size, the better the supercritical fluid interacts with the plant matrix, thus increasing the extraction yield [51,52,53,54,55]. At the end of the process, it must be ground to a certain size to expand the surface area. After the grinding process, the plant is passed through sieves with certain pore sizes and the particle size of the plant accumulated at the bottom can be calculated if desired [33]. After all the processes, the plant material should be placed in dark bottles to avoid being affected by light and stored in a deep freezer between −4 and −20 °C until the extraction process.

**Table 5:** Extraction examples using ScCO_2_ (2018–2024 literature summary).

Entry	Plant Name (Plant Material) [Region]	Bioactive Compounds or Compound Classes	Temperature (°C)	Pressure (bar)	Time (min)	Co-Solvent	Flow Rate	Particle Size	Reference
1	*Lippia graveolens* (flowers and leaves) [Mexico]	Cirsimaritin, Quercetin, Phloridzin, Apigenin, Naringenin, Luteolin	58.4	166	75	12.44% Ethanol	5 mL/min	-	[29]
2	Bee pollen [Colombia]	β-carotene	60	280	360		5000 mL/min	-	[56]
3	*Camellia sinensis* L. (leaves) [Switzerland]	Carotenoids, Chlorophyll B, Chlorophyll A, Caffeine	35 and 70	300	90	Ethanol, ethanol/water (1:1) or water	-	0.34 mm	[26]
4	*Phaseolus vulgaris* L. (seeds) [Peru]	α-Tocopherol, β-Tocopherol, γ-Tocopherol, δ-Tocopherol	35–45	380–420	30–120	-	1 mL/min	-	[57]
5	Tobacco (rhizomes) [Zimbabwe]	Nicotine	45–65	250	60–120	Ethanol	-	0.35–0.42 mm	[28]
6	*Cosmos sulphureus* (seeds) [China]	Catechin, Epicatechin, Coreopsin, Sulfuretin, Luteolin, Quercetin, Butein, Myricetin, Rutin, Hesperidin, Kaempferol, Naringenin	55	250	75–105	10% Ethanol	0.5 g/min	-	[27]
7	*Rosmarinus officinalis* L. (leaves) [Germany]	Quinic acid, Danshensu, Hydroxybenzoic acid, Caffeic acid, Gallocatechin, Rosmarinic acid, Luteolin-*O*-glucuronide, Salvianolic acid A, Luteolin-*O*-acetylglucuronide I, Luteolin-*O*-acetylglucuronide II, Cirsimaritin, Ladanein, Rosmanol, Epirosmanol, Isorosmanol, Genkwanin, Epiisorosmanol, Epirosmanol methyl Ether, Methoxyrosmanol, Carnosol, Rosmadial I, Rosmadial II, Carnosic acid I, Carnosic acid methyl Ester, Carnosic acid II	40, 50 and 60	100	40	-	7000 mL/min	-	[46]
8	*Lippia origanoides* [Colombia]	Taxifolin-hexoside, Quercetin-3-glucoside, Luteolin deoxyhexoside, Eriodictyol hexoside, Luteolin hexoside, Taxifolin, Galangine-hexoside, Phloridzin, Eriodictyol, Quercetin, Luteolin, Naringenin, Hesperetin, Chrysoeryol, Cirsimaritin, Sakuranetin, Pinocembrin, Dimethylated flavone, Trimethylated tricetin, Galangin, Methylated galangin	40	300	180	89% *v*/*v* aqueous ethanol	39 g/min	-	[43]
9	*Cymbopogon flexuosus* (leaves and stems) [Palestine]	Hydrocarbon monoterpenes, oxygenated monoterpenoids, hydrocarbon sesquiterpenes	40	150	15	-	200 g/60 min	-	[44]
10	Quebec LSD-type Cannabis (flowers) [Canada]	Tetrahydrocannabinol (∆ 9-THC), Cannabigerol (CBG), Cannabinol (CBN)	40–70	150–320	120–240	-	5–15 g/min	-	[58]
11	*Maackia amurensis* (bark) [Russia]	Flavones, Isoflavones, Flavonols, Flavonoids, Isoflavans, Prenyl flavonoids, Phenolic acids, Coumarins,	31–70	50–400	60–90	2% Ethanol	10–25 mL/min	-	[59]
12	*Ledum Palustre L.* (Leaves and twigs) [Russia]	Flavanols, Flavones, Flavan-3-ols, Coumarins Terpenes, Terpenoids	60	350	60	3.5% Methanol	-	-	[60]
13	*Quercus infectoria* (gall) [Indonesia]	Phenolic compounds, Coumarins	60	200	60	0.05% Methanol	25 mL/min	0.3 mm	[38]
14	*Terminalia chebula* (pulp) [India]	(+)-Chebulic acid, Gallic acid, Ellagic acid, Quercetin 3-O-glucuronide, Asiatic acid, Fertaric acid, Amlaic acid, Isoterchebin, 2-Hydroxychromene-2-carboxylate, 2,6-Digalloylglucose, a-Santalal, Dambonitol, Terbinafine, Tiropramide, Solanocapsine, Erythromycin B, Telaprevir, Nafoxidine, Novaluron, Dihydrodeoxystreptomycin, Sphinganine, Stigmatellin Y, Oxymetazoline, l-Arginine, Retronecine, S-(Hydroxymethyl) Mycothiol, Oxdemetonmethyl, Punicacortein B,	51.97	166.94	67.47		3.34 mL/min	0.149 mm	[30]
15	*Ziziphus jujuba* L. (fruits) [Iraq]	Andrographolide, Spiro[androst-5-ene-17,1′- cyclobutan]-2′-one, 3-hydroxy-, (3β,17β)-, 1,3-Bis-t-butylperoxy-phthalan, Cyclobarbital, Androstane-11,17-dione, 3-[(trimethylsilyl)oxy]-, 17-[O- (phenylmethyl) oxime], (3α,5α)-	52	270	240	90% ethanol	50 g/min	-	[45]
16	*Narcissus poeticus L.* (Flowers) [Lithuania]	Monoterpene hydrocarbons, Oxygenated monoterpenes, Oxygenated sesquiterpenes Diterpenoids, Triterpenoids, Tocopherols, Phenylpropanoids, phenols	40	480	10	5% (*v*/*v*) ethanol	2000 mL/min	0.5 mm	[61]
17	*Capsicum annuum* L. [Hungary]	Carotenoids	50	350–450	60	-	15,000 g/60 min	<0.2 mm	[40]
18	*Lamium album* (white dead nettle, *Lamiaceae)* (Flower) [Poland]	Phenolic compounds,	40–60	250	180	25% methanol	4 mL/min	-	[47]
19	*Elaeagnus mollis* Diels (seeds) [China]	Amino acids	37	300–310	210	-	-	0.22 mm	[62]
20	*Iberis amara* (seeds) [China]	Cucurbitacin E	40–60	350	20–100	3–15% ethanol	1–3 mL/min	0.45 mm	[63]
21	*Eugenia uniflora* (leaves) [Brazil]	Selina-1,3,7(11)- trien-8-one, Phytol, Phytol Acetate, Vitamin E, γ-sitosterol, Vitamine C, Friedelin	40–60	150–250	90	-	4 mL/min	0.204 mm	[64]
22	*Vitis vinifera* L. (leaves) [Portugal]	α-tocopherol, β-sitosterol, β-amyrin, Lupeol	40–80	300	30–360	Ethanol 5–10 wt.% Ethylacetate 5–10 wt.%	12 g/min	30 mm	[65]
23	*Cnidoscolus quercifolius* (seeds) [Brazil]	Tocopherols, β-sitosterol	40–60	200–300	120		5 g/min	0.51 mm	[66]
24	*Beta vulgaris* (roots) [Argentina]	Polyphenols	35–50	300–400		75% ethanol	600 g/60 min	-	[67]
25	*Carum copticum* L. and *Thymus vulgaris* L. (seeds) [Egypt]	Monoterpenes hydrocarbons, Oxygenated monoterpenes, Sesquiterpenes hydrocarbons, Oxygenated sesquiterpenes, Thymol	40	104 and 167	90	-	8 mL/min	0.5 mm	[68]
26	*Lippia graveolens and Lippia* *origanoides* (leaves) [Colombia]	Taxifolin, Eriodictyol, Quercetin, Luteolin, Naringenin, Hesperetin, Apigenin, Chrysoeriol, Dimethylated flavone, Pinocembrin, Cirsimaritin, Sakuranetin, Galangin, Methylated galangin, Methylated apigenin	40–60	80–400	60–120	10% ethanol	10–50 g/min	0.5–2 mm	[69]
27	*Camellia sinensis var. assamica,* (seeds) [ Thailand]	Flavonoids, Phenolics, Saponins, Tannins	40–60	125–250	60–300	-	110–170,000 mL/60 min	-	[48]
28	*Morus alba* (leaves) [Brazil]	β-sitosterol, Phytosterol, Phenolics	40–60	150–200	120	-	2 g/min	0.297–0.71 mm	[70]
29	*Rhododendron sichotense Pojark*. and *Rhododendron adamsii* Rheder (leaves and stems) [Russia]	Flavonoids, Phenolics	50–60	300–400	60–70	1% ethanol	50 mL/min	-	[71]
30	*Bauhinia forficata subsp.* *pruinosa* (leaves) [Brazil]	β-sitosterol, γ-tocopherol, α-tocopherol, Phytol, Lanosterol	40–60	180–220	200	-	2 mL/min	0.297–0.71 mm	[72]
31	*Ziziphus jujuba Mill. cv. Junzao* (leaves) [China]	Quercetin 3- *O*-robinobioside, Rutin, Hyperoside (quercetin-3- *O*-D-galactoside), Quercetin-3- *O*-D-glucoside, Kaempferol-3-*O*-robinobioside, Kaempferol-3-*O*-glucoside, Quercetin-3-*O*-β-L-arabinosyl-(1→2)-α-L-rhamnoside, Quercetin-3-*O*-β-D-xylosyl-(1→2)-α-L-rhamnoside	45–55	200–300	60–120	90% ethanol	0.2–0.6 mL/min	0.25 mm	[34]
32	*Salvia fruticosa* (leaves) [Greece]	Monoterpenes, Oxygenated monoterpenes, Sesquiterpenes, Oxygenated sesquiterpenes, Diterpenes	40–60	100–280	150	-	1–3000 g/60 min	0.2 mm	[73]
33	*Senecio brasiliensis* (leaves, flowers, and stalks) [Brazil]	β-elemene, α-humulene, Caryophyllene, Germacrene-D, Bicyclogermacrene, Spathulenol, Neophytadiene, Phytol, Isospathulenol, Senecionine, Integerrimine, Vitamin E	40–60	150–250	45	-	4 g/min	1.29 mm (leaves) 1.07 mm (flowers 0.802 mm (stalks)	[74]
34	*Hibiscus sabdariffa* (flowers) [Spain]	Quercetin-3-glucoside, Methylepigallocatechin, Myricetin, Quercetin, Kaempferol, Phenolics	40–60	150–350	90	7–15% ethanol	25 g/min	2 mm	[75]
35	*Bixa Orellana* L. (seeds) [Brazil]	Tocotrienols, Tocopherols, Geranylgeraniol	40–60	100–310	100–115	-	5 g/min	3.4 mm	[35]
36	*Chrysopogon zizanioides* (roots) [Brazil]	Cis-eudesm-6-en-11-ol, Khusimone, Maaliol, Epi-zizanone, Zizanol, Khusiol, Spathulenol, Vetiselinenol, Khusimol, Isovalencenol, Nootkatone, α-Vetivone, Isovalecenal, β-Vetivone, Zizanoic acid	40–60	140–200	150	1%, 3% or 5% (*v*/*v*) ethanol or ethyl acetate	1.97 g/min	0.45 mm	[36]
37	*Tiliae Inflorescentia* L. (flowers) [Poland]	Tiliroside, Eriodictyol, Quercetin, Apigenin, Naringenin, Kaempferol, Isorhamnetin, Sakuranetin	45–80	100–220	20–60	5% or 10% ethanol	10.33 mL/min for 5% ethanol 13.65 mL/min for 10% ethanol	-	[76]
38	*Arctium lappa* (leaves) [Brazil]	Phytol acetate, Phytol, ϒ-sisterol, Lupeol acetate, α-amyrim, Amyrin acetate, Lupeol	40–80	150–250	25–150	33% ethanol	2 mL/min	-	[37]
39	*Eugenia pyriformis Cambess*. (leaves) [Brazil]	Vitamin E, β-Sitosterol, β-amyrin, α-amyrin	40–60	100–200	210	-	2 g/min	0.73 mm	[77]
40	*Ocimum basilicum* L. (leaves and flowers) [Portugal]	Phenolics, Flavonoids, Terpenes	40	400	150	-	1000 g/60 min	0.6 mm	[78]
41	*Malus pumila* (seeds) [New Zealand]	Phenolics	40	Up to 1300	300	-	6–10 mL/min	0.5 and 1 mm	[33]
42	*Populus nigra* L. (buds) [Poland]	Phenolics, Monoterpenes, Flavonoids, Sesquiterpenes, Diterpenes	40–60	83–337	60	-	2000 g/60 min		[79]
43	*Ruta Chalepensis* L. (roots) [Turkey]	Coumarins, Furanocoumarins, Phenolics, Furano alkaloids, Quinoline alkaloids, Acridanone alkaloids	45	90 and 200	50	-	2 mL/min	-	[80]
44	*Pterocaulon polystachyum*	Coumarins	60	240	120	-	1000 g/60 min	-	[81]
45	*Juglans regia* L.	Linoleic acid, Oleic acid, Palmicitic acid	59.85	400	315–390	-	-	-	[82]

### 1.5. Characterization and Isolation Methods

Fractionation, which can be performed during or after extraction, separates the ScCO_2_ extract into fractions with similar properties. This process is often achieved by adjusting the pressure and temperature during extraction or using a series of separators operating at different pressures. Fractionation is usually applied to plant extracts such as green tea [83], ginseng root [84], camphor tree [85], eucalyptus [86], and others to isolate and purify specific compounds or groups of compounds.

For example, in the studies of black and green tea, fractionation was used to isolate and determine different classes of chemical compounds by using the column solid phase extraction (SPE) method to separate the elements into hydrophobic (HF), cationic (CF), and residual (RF) fractions. Subsequent determination of the concentrations of aluminum (AI), barium (Ba), calcium (Ca), iron (Fe), magnesium (Mg), manganese (Mn), and nickel (Ni) in the separated columns of eluates was carried out using inductively coupled plasma optical emission spectrometry (ICP-OES) [83]. This approach allowed researchers to determine and compare the digestibility of elements from tea infusions and clearly differentiate and categorize tea samples by type using the principal component analysis (PCA) and linear discriminant analysis (LDA) methods.

Also, in 2019, Shukla et al. conducted a study demonstrating the effectiveness of ScCO_2_ extraction combined with online fractionation. They used this technique to efficiently extract volatile oil and oleoresin from dried ginger, enriched with essential bioactive compounds [87]. The need to investigate optimal parameters for oil and oleoresin extraction arose from the limitations of traditional methods [88]. Classical methods have high operating temperatures, which can cause the degradation of the key active compounds and worsen their quality. In addition, it is noted that using organic solvents such as acetone, trichloromethane, ethanol, hexane, isopropanol, and others can be hazardous to health, as some of them may have a carcinogenic effect [89].

In the study [84], fractionation was used to isolate and characterize water-soluble polysaccharides from *Panax ginseng* roots. This practical process was crucial for obtaining different fractions of polysaccharides; it allowed the separation of water-soluble polysaccharides into two neutral fractions and six acidic fractions using a combination of ethanol precipitation, ion exchange chromatography, and gel permeation chromatography methods. As a result, components such as starch-like glucans and pectins rich in different sugars and acids were isolated, which allowed a more detailed study of their structure and immunological activity. Thus, fractionation served to obtain homogeneous samples of different polysaccharides from ginseng, a critical step for subsequent analysis of their structure and functional properties and potentially leading to the development of new drugs or functional foods [84]. Zhang et al. used fractionation to separate components, analyze composition, and assess biological activity. Specifically, the researchers used various chromatographic techniques to isolate neutral and acidic polysaccharide fractions and ginseng extracts, resulting in pure fractions that could be analyzed separately. Individual fractions were then tested for their biological activity, such as the ability to stimulate lymphocyte proliferation [85].

In general, fractionation allows for a detailed study of the composition of complex biological extracts and the identification of active components. This is important for their potential use in medicine and the food industry.

#### 1.5.1. Chromatography

High-performance liquid chromatography (HPLC) is one of the most commonly used methods for purifying liquid plant extracts. HPLC can separate, identify, and quantify compounds within a mixture. It is commonly used to analyze plant extracts containing phenolic compounds, terpenes, alkaloids, and other biologically active substances. HPLC allows for the determination of low-concentration components in plant materials and ensures the purity of the results [90]. HPLC is used for such plants as ginseng (*Panax ginseng*) [91], blueberry (*Vaccinium myrtillus*) [92], turmeric (*Curcuma longa*) [93], green tea (Camelia sinensis) [94], and echinacea (*Echinacea purpurea*) [95]. This is only a small list of the plants suitable for analysis using HPLC. *Piper amalago* L. and Korean Ginseng extracts were obtained using ScCO_2_ chemicals and their chemical composition was determined using HPLC [96,97]. In another study, phenolic compounds were isolated from *Berberis vulgaris* fruit by using the ScCO_2_ extraction method. Potent antioxidant compounds such as rutin and apigenin were determined by HPLC [98].

In the study [91] related to the mechanism of the anticancer action of black ginseng, HPLC was used to determine the composition of black ginseng at different stages of its processing. Using HPLC, various chemical components were identified and quantified, including organic acids, maltol, 5-hydroxymethylfurfural (5-HMF), ginsenosides, amino acids, and oligosaccharides. This method allowed them to identify six small molecular weight organic acids, maltol, 5-HMF, 17 ginsenosides, four oligosaccharides, and 20 amino acids in the composition of black ginseng. Such key components were used in network pharmacology to study the mechanisms of the anticancer action of black ginseng. These results also formed the basis of the entropy weighting analysis method and network pharmacology methodology to explore the pathways and targets associated with the anticancer action.

In the article [92], HPLC-MS/MS was used to simultaneously determine 36 phenolic compounds in blueberries and strawberries and in their commercial products such as jam. The aim of finding a new method was to rapidly and comprehensively determine chemical compounds for the quality control and authenticity of berries and products based on them. Their work with HPLC-MS/MS included the optimization of parameters to achieve the best separation and sensitivity of the analysis. Then, samples were prepared with a mixture of ethanol and water, followed by centrifugation and filtration before injection of HPLC-MS/MS. In positive and negative modes, HPLC with gradient elution and electrospray ionization (ESI) were used to separate and quantify phenolic compounds. When applying the qualitative and quantitative analysis methods, about 36 phenolic compounds were determined, such as anthocyanins, flavanols, dihydrochalcones, and enolic acids. This method has proven reliable for quality control of berries and berry-based products.

Some sources also use it to determine curcuminoids in turmeric powder. It allows for the measurement of very low concentrations of curcuminoids, ensuring the accurate quantification of each component. In the study, HPLC contributed to the identification of the most effective methods for powder preparation [93].

The method has a high cost and uses organic solvents, which can be hazardous to health and the environment.

#### 1.5.2. Gas Chromatography (GC)

For extracts that can be vaporized without decomposition, GC is used. It is particularly beneficial for volatile bioactive compounds. For instance, numerous studies have utilized this method to extract lavender essential oil, which contains volatile components such as linalool and lavandula oil [99,100]. GC-MS combined with SPME (Solid Phase Microextraction) is an effective analytical approach for the separation of volatile organic compounds from lavender plant material [101]. In addition, this method is particularly valuable for assessing the quality of essential oils (EOs) due to their sensitivity [102]. Its high accuracy and sensitivity make this method essential in the pharmaceutical, perfumery, and food industries. Multiple examples of mint extract are analyzed by gas chromatography [103,104]. This method is suitable for assessing the purity and composition of volatile bioactive compounds that can be vaporized without undergoing decomposition.

There are many gas chromatography types and models; in the study [92], portable gas chromatography was used. The authors’ main goal was to quickly determine volatile organic compounds emitted by lavender and lavender plants in the field. The study used a portable GC-MS Torion T-9, which allows for rapid analysis directly on site without the need to transport samples to the laboratory. This reduces the analysis time and the risk of sample degradation.

GC was used to analyze the phytochemical components of *Mentha longifolia* ScCO_2_ extracts. The aim was to identify the active phytochemical components of different plant extracts with anticancer effects. Chemical analysis revealed significant amounts of fatty acids, phytosterols, and terpenoids [105].

GC-MS is also used to analyze the extract of *Nigella sativa* L. oil. The main component was thymoquinone. They were identified as having antioxidant potential; using the DPPH method, it was possible to confirm the significant medical potential of *Nigella sativa* L. oil [106].

#### 1.5.3. Counter-Current Chromatography (CCC)

This separation technique uses two immiscible liquid phases as the stationary and mobile phases. CCC does not require a solid stationary phase, eliminating the problems associated with its possible degradation and contamination [107]. It is used in the pharmaceutical industry to purify natural compounds and analyze food. In a study (Lu et al., 2007), the CCC method was used to isolate and purify three prenylflavonoids from *Artocarpus altilis*. In the first step of the two-dimensional counter-current chromatographic system, a vertical CCC instrument with a total capacity of 1600 mL was used to isolate the target compounds. These compounds were then collected in a 30 mL loop using a column switching system prepared in the laboratory and injected into a second high-speed column with a capacity of 210 mL for further separation. Two-phase solvents based on n-hexane-ethyl acetate-methanol-water were selected to optimize the separation in the ratios 5:5:7:3 and 5:5:6.5:3.5. These systems ensured the efficient separation and purification of prenylflavonoids [108].

In this paper [109], scientists used high-performance counter-current chromatography (HPCCC) to isolate and purify curcuminoids from *Curcuma longa* L. This study aimed to optimize the extraction conditions and test the retention of their biological activity after purification. The best extraction condition was 80% ethanol, an extraction temperature of 70 °C, a liquid-to-material ratio of 20:1, and an extraction time of 3 h. Under these conditions, the yield of curcumin was achieved at 56.8 mg/g. Then, using the solvent system of n-hexane/ethyl acetate/methanol/water in a ratio of 2/3/3/1, the researchers successfully isolated curcumin, demothoxycurcumin, and bisdemethoxy-curcumin. A total of 67 mg curcumin, 18 mg dimethoxy curcumin, and 9.7 mg bis(dimethoxy) curcumin were obtained with a purity of 98.26%, 97.39%, and 98.67%, respectively. Thus, the study demonstrated that the HPCCC method effectively isolates and purifies curcuminoids from turmeric, ensuring a high purity and maintaining the biological activity of the compounds.

In a study by Cai et al. (2021), the researchers used HSCCC to isolate and purify flavonoids from a *Malus hupehensis* extract obtained using deep eutectic solvents (DES). This method successfully separated five flavonoids, including two new compounds, 6′-O-coumaroyl-2′-O-glucopyranosylphloretin and 3″-methoxy-6′-O-feruloyl-2-O-glucopyranosylphloretin, as well as three known compounds, avicularin, phloridzin, and siboldin. The study also utilized a more environmentally friendly type of solvent, which consists of a class of liquid salts known for their high extraction capacity and low toxicity [110].

Long et al. optimized an efficient method based on ScCO_2_ and HSCCC has been developed for the extraction and preparative purification of polymethoxyflavones from Citri reticulatae pericarpium. Nobiletin, 3,5,6,7,8,3′,4′-heptamethoxyflavone, and tangeretin were obtained [111]. In another study, SFE and HSCCC were combined to extract and purify homoisoflavonoids from *Ophiopogon japonicus* (Thunb.) Ker-Gawler. methylophiopogonanone A 6-aldehydo-isoophiopogonone A and 6-formyl-isoophiopogonanone A were obtained successfully [112].

#### 1.5.4. Preparative Thin-Layer Chromatography

TLC isolates and purifies small quantities of compounds. It is a more straightforward and more cost-effective technique than column chromatography. In this method, layers of no more than 750 μm of the analytes are used, as thicker layers worsen the separation of chemical components [113]. This means that the thickness of the layer determines the limits of TLC’s capabilities. It should be noted that TLC in thin layers cannot fully compete with column chromatography. The procedure for conducting thin-layer chromatography typically includes several vital stages: dissolving the analyte in an appropriate solvent, applying the resulting solution onto a chromatographic plate, developing the plate in a suitable solvent system, and detecting the zones using ultraviolet light or chemical reagents [114]. This method is highly effective for performing qualitative and quantitative analysis in chemical-toxicological contexts and other areas where the precise and prompt determination of chemical compounds is required [115].

In another study, the aim was to evaluate the presence of a quantitative content of bioactive compounds in ScCO_2_ leaves extract of *Centella asiatica* [116]. The different Rf values suggest that these substances have different polarities, which is significant for their isolation and identification using other chromatographic and spectroscopic methods.

In a study by Bako et al., TLC was utilized to assess the potential antibacterial effects of *Salvia sclarea* L. [117]. This method enabled the researchers to determine the composition of the samples. TLC is a high-throughput screening technique that combines thin-layer chromatography with biodetection. It is cost effective, minimizes solvent usage, and allows for the simultaneous analysis of multiple samples under the same conditions [118]. TLC in combination with the GC-MS method was also used by Kazakh scientists for an experimental study of the effect of osmotic and salt stress and the effect of a low positive temperature on the metabolomes of immature medical plants *Sedum hybridum* L. [119].

### 1.6. The Use of Herbal Compounds in the Treatment of Cancer

#### 1.6.1. Clinical or Preclinical Study Examples

The use of herbal substances in cancer treatment is a significant research topic, with both preclinical and clinical trials being conducted [120]. Preclinical research frequently includes cellular and animal model trials to determine whether prospective herbal substances have anticancer properties, such as reducing cancer cell growth, inducing apoptosis, or inhibiting metastasis [121,122]. These studies, which are frequently undertaken in a laboratory setting, are a critical stage in the process of discovering and developing new anticancer drugs [123].

The clinical trials, on the other hand, are conducted on human subjects and provide information regarding the efficacy and safety of herbal substances for the treatment of cancer [124]. These studies typically explore the feasibility of utilizing herbal components in a specific form of cancer to mitigate cancer symptoms, impede disease advancement, or regulate the proliferation of cancer cells [125]. Clinical trials also assess the efficacy and potential adverse effects of herbal substances when used in conjunction with other cancer therapies, such as chemotherapy and radiotherapy [126].

Herbal substances extracted using ScCO_2_ have not been investigated for their potential in cancer treatment in any published clinical trials. The following is a summary of preclinical research that shows how extracts made by ScCO_2_ extraction can be used to treat cancer.

To investigate the selective cytotoxic activity of the leaf juice (FDLJ) of the *Carica papaya* plant grown in Australia on squamous cell carcinoma (SCC25) and the immortalized keratinocyte cells (HaCaT), ScCO_2_ extraction was performed under high pressure (250 bar), low temperature (35 °C), a long processing time (180 min), and a large amount of starting material (5 g). The cytotoxicity of leaf juice was found to be higher against the SCC25 cell line compared to HaCaT cells, according to the investigation conducted using the MTT method. Preliminary qualitative research suggests that vitamins and phytosterols are the primary components of the leaf juice content, and it is believed that these substances are responsible for the cytotoxic action [127].

Artepillin C (ARC), a prenylated phenolic compound found in Brazilian green propolis, has been shown to be effective as an anticancer nutritional supplement due to its capacity to inhibit tumor development and metastatic spread. The new approach combines adsorption chromatography and ScCO_2_ extraction to achieve the high purification of ARC. The optimal supercritical conditions for the CO_2_-ethanol extract were set at 4000 psi and 50 °C. Purified ARC considerably dispersed cells in the S phase and, based on its cytotoxic effects in human colon cancer cells (HCT116), potently suppressed cancer cell division compared to commercial ARC. ARC also inhibits the migration of HCT116 cells, demonstrating its capacity to limit tumor metastasis [128].

The triterpenoid extraction process was carried out using ScCO_2_ from the Vietnam *Ganoderma lucidum* (G. lucidum) plant. The optimal extraction conditions were found to be 37.5 MPa pressure, 48 °C extraction temperature, and 1.5 h of extraction time using the Box–Behnken design. Diverse analytical methodologies have confirmed the existence of triterpenoids. The cytotoxic effect of *G. lucidum* extract on KB (carcionama cells), HepG2 (liver cancer cells), Lu (lung cancer cells), and MCF-7 (breast ancer cells) cancer cells (IC_50_: 90.35–162.13 μg/mL) makes it a viable candidate for use as an anticancer agent. Because the extract includes triterpenoids, polysaccharides, phenolics, steroids, and alkaloids—all of which are recognized for their different anticancer effects—its anticancer effect makes sense [129].

The anticancer properties of *Cannabis* flower extracts (2,3,4,5) were assessed using various extraction methods, including ScCO_2_ with and without modifiers. The extracts were then tested against colorectal adenocarcinoma (Caco-2), prostate cancer (PC3), and cervical cancer (HeLa, SiHa and C33) cell lines to determine their effectiveness. Additionally, their cytotoxicity against HaCat, African green monkey kidney epithelial cells (Vero), and fibroblast L929 (L929) cells was evaluated. Among the extracts, extract 5 (obtained through a 30 min preheating at 140 °C, 40 MPa, and 50 °C) exhibited the highest activity against all cancer cell lines. In addition, extract 5 showed a higher activity against Hela, SiHa, and C33 cell lines than other cancer lines, while extracts 2 (30 min 140 °C preheating, 40 MPa, 70 °C), 3 (120 min 140 °C preheating, 22 MPa, 70 °C), and 4 (120 min 140 °C preheating, 40 MPa, 50 °C) showed the lowest cytotoxicity against HaCat, Vero, and L929 cell lines [130].

Mango seed kernel (MSK) is a rich source of bioactive phenolic compounds that have been found to exhibit antiproliferative effects on colon cancer cell lines. The extraction process involves two steps, sequential pressurized liquid extraction (PLE) followed by supercritical antisolvent fractionation (SAF), to enhance the anticancer activity of the extract against the colorectal adenocarcinoma (HT-29) cell line. In SAF, pure ScCO_2_ is used along with a water/ethanol mixture. In the method optimized using the Box–Behnken experimental design, the highest cell proliferation inhibition (70.51 ± 1.14%) was obtained using 50% water *v*/*v* in the feed extract and a feed/ScCO_2_ ratio of 0.0625 at 15 MPa. The LC-q-TOF-MS/MS analysis of the phytochemical profile revealed a strong correlation between the antiproliferative activity and the presence of xanthones and gallic acid derivatives [131].

To explain the relationship between the antioxidant activity of *Baccharis uncinella* supercritical extract and its fractions obtained under different conditions, anticancer activity against bladder (T24) and glioblastoma (U87) cell lines, and cytotoxic activity against healthy cells (Vero), the extracts were obtained using ethanol and ScCO_2_ (150 and 200 bar and 60 °C). The methanol fraction showed 86.7% antioxidant activity, close to the standard antioxidant quercetin used in the test (87.9%). The methanol fraction reduced cell viability to less than 50% at concentrations of 158.8 μg/mL in T24 cells, 213.9 μg/mL in U87 cells, and 702.5 μg/mL in Vero cells. The results indicate that the methanol fraction of the supercritical extract of *B. uncinella* exhibited an inhibitory effect on the growth of the examined tumor cells, whereas its impact on normal cells was comparatively weaker [132].

To maximize the flavonoid production from Xinjiang jujube leaves (XJL), ScCO_2_ liquid extraction (SFE−CO_2_) and conventional extraction conditions were evaluated; SFE-CO_2_ produced the highest flavonoid content. Response surface methodology (RSM) was used to optimize the SFE-CO_2_ conditions, and the Box–Behnken design (BBD) was used to assess how four factors affected the flavonoid output. The optimal conditions to obtain the highest flavonoid yield were determined as 52.52 °C temperature, 27.12 MPa pressure, 113.42 min time, and 0.44 mL/min co-solvent flow rate, and under these conditions, the flavonoid yield was obtained as 29.052 ± 0.38 mg/g. In a comparison of the antiproliferative activities of extracts obtained using various methods, SFE−CO_2_ (64.17 ± 0.45%) extracts outperformed conventional Soxhlet extraction (33.72 ± 0.48%) and ultrasound-assisted extraction (41.32 ± 0.77%) against the lung adenocarcinoma (A549) cell line [34].

The potential antitumor properties and antiproliferative, autophagic, and apoptosis-inducing effects of *Croton crassifolius* essential oil (CCEO) obtained by ScCO_2_ extraction (25 MPa at 35 °C 30 min dynamic extraction with a flow rate of 2 L CO_2_/min) were evaluated on five cancer cell lines (A549, HeLa, HepG2, T24, and gastric cancer (MGC803)) and one normal human cell line (HL-7702) via CCK-8 assays, indicating that CCEO had a remarkable effect with significant selectivity on all cancer cell lines. CCEO showed the best activity (IC_50_: 25.00 ± 1.62 μg/mL), especially against the A549 cancer cell line. CCEO inhibited cell proliferation and colony formation in A549 cells in a dose- and time-dependent manner. CCEO also demonstrated that it reduces the expression of cyclin B1-CDK1 and cyclin A-CDK1 in the G2/M phase while raising the expression of cyclin-dependent kinase inhibitor (CKI) P21 at both the transcriptional and translational levels. Autophagic staining assays and Western blot analysis revealed that CCEO induced the formation of autophagic vacuoles in A549 cells and increased the expression of autophagy-related proteins beclin-1 and LC3-II in a dose-dependent manner [133].

Potential anticancer effects were observed when the extracts of *Helichrysum italicum* produced by extraction using ScCO_2_ were assessed against the cancer cell lines MCF-7, HeLa, and human fetal lung fibroblast cells (MRC 5). There were notable cell line-specific effects on NF-kB activation at extract concentrations lower than IC_50_ values. The different responses of HeLa and MCF-7 cells indicate the importance of cell receptors for biological activity [134].

The extraction of ginger extract, which contains high levels of terpene hydrocarbons, oxygenated terpenes, and other non-volatile chemicals, was performed using ScCO_2_ extraction. The extraction conditions included a pressure of 35 MPa, a temperature of 35 °C, a CO_2_ flow rate of 15 L/s, and an extraction period of 2 h. The extract’s cytotoxic effects, as well as the distillates generated from molecular distillation at various temperature and pressure conditions, were assessed against breast cancer (MDA-MB-231), A549, and HepG2 cell lines. Ginger extract and its distillates strongly inhibited the proliferation of A549, while 6-gingerol and zingerone-rich distillate (IC_50_; MDA-MB-231: 63.74 ± 1.56 μM, A549: 60.13 ± 2.19 μM, HepG2: 69.84 ± 1.82 μM) showed the best cytotoxicity against all cell lines [135].

The main fraction of the chemical composition of the extract obtained from *Pistacia lentiscus L.* leaves with ScCO_2_ was terpenes, and the most abundant molecules were determined as Germacrene D (11.18%), delta-cadinene (10.54%), and alpha-pinene (8.7%). Cell proliferation and cell toxicity were unaffected by increasing extract concentrations in endothelial cells (ECs). The extract demonstrated dose-dependent antioxidant activity in non-stressed and H_2_O_2_ treated ECs [136].

The optimization of ScCO_2_ extraction conditions for *Arnica montana* L. flower heads, which are recognized for their anticancer activity, resulted in a high extraction efficiency and a significant content of biologically active components. The optimized conditions included a temperature of 60 °C and a pressure of 30 MPa. The extract exhibited an anticancer activity of up to 85% against metastatic melanoma WM266-4 cells when tested at a dosage of 0.5 mg/mL [137].

The Neem tree (*Azadirachta indica*), renowned for its anti-inflammatory properties, contains a high concentration of liminoid terpenoids, specifically, azadiractoids. In order to examine the impact of this herb on colorectal cancer, human HCT116 and HT29 cells were administered ScCO_2_-derived extract (SCNE) or neem liminoid or nimbolide. The treatment with SCNE resulted in an increase in apoptosis and a dose-dependent decrease of CRC cell growth. The transcription factors STAT3 and NF-κB, which are involved in the control of genes for many cellular activities, had their expression decreased after being treated with SCNE and nimbolide. The Western blot and zymogram analyses demonstrated that the anti-invasive effects were observed through a reduction in the protein expression of MMP2 and MMP9 in CRC cells. Moreover, the treatment of mice with HT29 and HCT116 xenograft tumors showed a remarkable reduction in colon tumor growth. Additionally, an overview of the extraction conditions, herbal sources, and anticancer activities of ScCO_2_ extracts has been presented in Table 6 [138].

#### 1.6.2. The Significance of Herbal Compounds in Discovery of New Drugs

Early people not only used plants as a source of nutrition, but they also realized that certain plants could be used to heal a variety of illnesses [139,140]. These therapeutic herbs, discovered via trial and error, were documented and passed down from generation to generation with the introduction of writing [141]. Clay tablets discovered in Mesopotamia provide evidence of the utilization of therapeutic herbs around the period of 2600 B.C [142]. The Ebers Papyrus, an ancient written document, includes a total of 77 herbal cures and over 800 prescriptions [139]. Herbal remedies were extensively utilized in ancient China, India, Greece, and the Middle East [143]. The Vedas, the sacred texts of Hinduism, have references to the usage of herbal remedies throughout India [144]. Hippocrates used plants to cure a wide range of ailments in ancient Greece, classifying them based on their healing properties [145].

Currently, substances derived from plants are thoroughly scrutinized through chemical and biological investigation [146]. A variety of state-of-the-art methods are employed to ascertain the molecular composition and investigate the biological effects of herbal compounds, including gas chromatography-mass spectrometry (GC-MS), liquid chromatography-nuclear magnetic resonance (LC-NMR), and GC-MS [147]. Plant extracts can be evaluated against biological targets thanks to high-throughput screening (HTS) technology. These approaches allow for rapid testing of hundreds of plant extracts, with the ability to separate extracts with biological activity by chromatography and identify active compounds [148].

Pharmacognosy is a scientific discipline that focuses on analyzing the chemical constituents of plants and investigating their biological impacts [149]. Although ethnopharmacology explores herbal remedies used in various cultures, research in both areas significantly advances the procedures involved in the discovery of new drugs [150]. Modern drug development still heavily relies on natural ingredients as a crucial resource [151]. Steroid hormones [152] and antibiotics [153] are plant-derived substances, along with cancer treatment drugs such as paclitaxel and docetaxel [154].

Herbal compounds serve not only as medicinal substances but also as nutritional supplements and functional foods [155]. Cruciferous vegetable glucosinolates [156], citrus fruit limonoids [157], soy isoflavones [158], and tomato lycopene [159] are just a few examples of the beneficial substances found in these foods. The discovery and application of natural compounds is aided by the identification of their biological activity and chemical profiling techniques [160]. Methods for isolating bioactive compounds from plant extracts include metabolic profiling, biotransformation-oriented techniques, biological activity-based fractionation, and synergy-oriented fractionation [161]. Herbal substances will still be used to treat a variety of illnesses including cancer [120], cardiovascular disease [162], diabetes [163], infections [164], and neurological problems [165] both now and in the future.

To summarize, plants and herbal substances have had a significant impact on the development of drugs throughout history and continue to do so in the present day. Contemporary scientific methodologies enable the efficient utilization of these substances, and this progression will persist in the coming times [166].

Natural products have historically been crucial in the process of drug discovery and have served as the primary reservoir of novel medications for numerous life-threatening illnesses. Many early medications were based on natural components, which are still a useful resource for illnesses for which there are no approved treatments [167]. Some natural substances derived from plants and their medicinal properties are enumerated below.

Phytochemicals of significance, such as curcumin derived from the *Curcuma Longa* plant, epigallocatechin-3-O-gallate extracted from the *Camellia sinensis* plant, and quercetin acquired from *Allium cepa*, are recognized for their antibacterial, anticancer, antidiabetic, antioxidant, and anti-inflammatory properties [49].

The treatment of Alzheimer’s disease involves the use of genistein, which is isolated from the *Genista tinctoria* plant [168,169], and Galantamine, a drug that is derived from *Galanthus caucasicus* [170,171]. Furthermore, the *Taxus brevifolia* Nutt. species is the source of the chemotherapy medication paclitaxel [172]. Natural compounds such as Podophyllotoxin (*Podophyllum emodi* Wall. and *P. peltatum* L.) [173], Lapachone (*Tabebuia avellanedae*) [174], Masoprocol (*Larrea tridentate*) [175], Lodopyridone (*Saccharomonospora* sp.) [176], and Salinosporamide A (*Salinospora tropica*) [177] are known to have anticancer properties.

#### 1.6.3. Application of Herbal Compounds in Cancer Treatment; Synergistic Effects and Combination Treatments

Cancer is globally recognized as the second most prevalent cause of mortality [178]. More time is required to manage cancer and its consequences even with great efforts towards cancer treatment [179]. Consequently, a significant number of individuals are turning to complementary and alternative medicine (CAM), which encompasses methods such as herbal drugs, homeopathic drugs, nutritional supplements, and anthroposophic medicine, as a result of the numerous side effects associated with chemotherapy, which is currently the primary therapy for cancer [180]. Although the use of complementary and alternative medicine (CAM) is increasing worldwide due to its proven ability to improve the life quality in a variety of patient populations, including cancer patients, it is important to note that there are several interactions between CAM and chemotherapeutic drugs. Herbal drugs interact with the liver’s metabolic system, particularly the cytochrome P450 system or phase-II metabolic system, which breaks down and activates drugs; this interaction is a major worry around the world because of the nature of chemotherapy treatments [181].

Patients undergoing chemotherapy have been observed to adopt alternative therapies, which have been shown to alleviate chemotherapy-related symptoms [182,183]. Some patients inform their doctors about their use of herbal remedies, but others do not, and many are unaware of the specific contents of the herbal remedies they take during chemotherapy [184]. There are pros and cons to using herbal medicines with chemotherapy, but the pros often outweigh the cons in managing chemotherapy-related side effects [185]. The combination of herbal medicines and chemotherapeutic agents can result in synergistic, antagonistic, or detrimental effects [186]. Therefore, it is advisable to choose and include natural pharmaceuticals that produce synergistic effects in chemotherapy [187]. Patients should receive advice on using natural products and be educated about the advantages and possible drawbacks of herbal remedies [188].

Natural products have recently gained a lot of interest as a potential solution to these problems. Cancer treatment stands to benefit greatly from the use of natural products due to their safety, efficacy, and cost-effectiveness. An important class of natural compounds, phytochemicals, have been used either alone or in conjunction with other medications to treat or prevent a variety of diseases [189]. Numerous mechanisms are responsible for the anticancer effects of phytochemicals, such as the induction of apoptosis, the modulation of cell signaling pathways, the arrest of the cell cycle, and the induction of DNA damage [190]. These compounds can help prevent tumor growth by blocking the generation of carcinogenic substances or the interaction of carcinogens with biomolecules. Several plant-derived anticancer drugs have been authorized for clinical application [191].

Curcumin derived from *Curcuma longa* is recognized for its ability to demonstrate anticancer properties by triggering apoptosis, impeding proliferation, and causing cell cycle arrest in several types of cancer cells [192]. In multiple animal models, organosulfur compounds from *Allium sativum* inhibited the progression of chemically generated cancers [193]. Breast cancer, advanced testicular cancer, lung cancer, lymphomas, leukemia, and Kaposi’s sarcoma are among the many cancers that have benefited from the synergistic use of *Catharanthus roseus* alkaloids vinblastine and vincristine with other anticancer medications [191,194,195]. Some phytochemicals can inhibit the NF-kB signaling system, which is essential to the development of cancer, whereas others, such as apigenin, crocetin, and quercetin, can reduce excessive activity of the MAPK/ERK signaling pathway. By enhancing apoptosis, punicalagin and esculetin ensure the death of cancer cells [190]. Phytochemicals such as ferulic acid and withaferin induce cell cycle arrest in cancer cells by blocking cyclin-dependent kinases, which regulate the cell cycle [196,197].

The anticancer properties of phytochemicals offer a promising approach to cancer treatment, and their combination with chemotherapeutics may result in more successful treatment regimens. For the clinical application of phytochemicals and their integration into cancer therapy, more epidemiological and preclinical research is needed. Progress in this domain has the potential to expand the range of therapeutic choices available to individuals with cancer and enhance treatment outcomes.

The synergistic effects of several anticancer drugs and significant plant extracts, flavonoids, and alkaloids have been well-documented. [187].

Research has demonstrated that curcumin and 5-Fluorouracil (5-FU), when combined, can sensitize cells of colon cancer that have become resistant to treatment, leading to a decrease in the amount of medication needed for treatment [198]. A considerable decrease in gastric cancer cell proliferation and an increase in the apoptosis index were seen when oxaliplatin was combined with 5-FU and curcumin [199]. Co-administration of curcumin and paclitaxel in the Xenograft mice model for breast cancer resulted in enhanced drug accumulation in tumor cells and reduced tumor development [200]. Curcumin also made paclitaxel more toxic to the breast cancer cell line MDA-MB-435 [201]. Greater inhibition was shown on PC-3 tumors when α-tomatin and curcumin were combined, as compared to when each drug was used alone [202].

Together, resveratrol and 5-Fu inhibited NF-κB activation, induced apoptosis, and modulated the TNF-β signaling pathway against HCT116 cancer and human colon cancer resistant (HCT116R) cell lines [203]. ROS burst and extracellular signal-regulated kinase-induced apoptotic cell death and cytoprotective autophagy were observed in cancer cell lines and a xenograft mouse model when resveratrol and temozolomide were co-administered [204]. Resveratrol, in combination with cisplatin, suppressed the apoptosis-dependent mechanism and glutamine metabolism in human hepatoma (C3A and SMCC7721) cell lines [205].

The coadministration of epigallocatechin-3-gallate and cisplatin resulted in an increase in the expression of CTR1 and enhanced the cellular absorption of cisplatin in human lung cancer cell lines [206]. By down-regulating the expression of the SOX2OT variant 7 gene, the combination of epigallocatechin-3-gallate and doxorubicin decreased autophagy in the osteosarcoma (U2OS and SaoS 2) cell lines [207].

The berberine alkaloid, in conjunction with cisplatin, enhanced the occurrence of apoptotic cell death in the MCF-7 cell line and controlled the mechanisms responsible for DNA repair [208]. Furthermore, the berberine alkaloid and cisplatin enhanced apoptotic and necroptotic cell death and inhibited growth against the ovarian cancer cell line (OVCAR3) [209].

Piperlongumin and paclitaxel enhanced reactive oxygen species (ROS)-induced apoptosis in human embryonic intestinal (INT-407) and HCT116 cancer cell lines [210]. In both in vitro and in vivo studies, piperlongumine decreased docetaxel efflux and enhanced bioavailability when administered to breast cancer and colon cancer cell lines in conjunction with docetaxel [211].

*Moringa oleifera*, in combination with 5-fluorouracil and cyclophosphamide, effectively inhibited the growth of hepatocarcinoma sarcoma in rats with hepatocarcinoma and resulted in an increase in body weight [212]. Together, cisplatin and *Morinda citrifolia* reduced cardiac damage in a rat model and showed cytoprotective activity against normal cells [213]. In leukemia and lung cancer animal models, the combination of *Marsdeniae tenacissimae* with paclitaxel and gefitinib considerably reduced tumor growth [214,215].

## 2. Conclusions and Future Perspectives

The use of the ScCO_2_ technique will have a growing significance in acquiring plant extracts due to its utilization of environmentally sustainable and non-hazardous solvents, as well as its ability to operate at low temperatures, thereby ensuring the preservation of bioactive components. Currently, this approach exhibits a reduced environmental footprint in comparison to extraction methods employing conventional solvents. Implementing recycling practices and employing CO_2_ use in the extraction process, rather than emitting it straight into the atmosphere, effectively decreases the carbon footprint and mitigates environmental degradation. Future technological developments on this approach will allow extraction tools to become more user-friendly and efficient. To optimize the extraction parameters and increase productivity, artificial intelligence and machine learning algorithms are going to be especially helpful. Purer and more bioavailable extracts can be obtained in this manner.

Biotechnology and genetic engineering are paving the way for plant extracts to be used as treatments for cancer and other health disorders. Unearthing novel plant species and harnessing extracts from genetically modified plants could yield more potent substances to combat certain forms of cancer. Furthermore, high-throughput screening methods and bioinformatics tools will enable us to better grasp the effects of these extracts on cancer cells and pinpoint the most efficient components, guiding the development of more focused and side-effect-minimizing therapy options in cancer treatment.

By means of expanding clinical investigations, we will be able to better grasp the safety and efficacy profile of plant extracts acquired with ScCO_2_ and facilitate the general use of these extracts in clinical settings. Enhancements in regulatory approval procedures will expedite the utilization of these extracts as medicinal substances. The creation of integrated and supportive therapy alternatives in cancer treatment would enhance patients’ quality of life and lower adverse effects connected to the treatment.

From an environmental standpoint, ScCO_2_ extraction makes good contributions to climate change with decreased energy usage and the use of ecologically friendly solvents compared to conventional methods, which is regarded as a crucial step in reaching sustainability goals. This technology is anticipated to alleviate chemical waste and hazardous emissions, particularly in large-scale industrial applications, and contribute significantly to the fight against environmental pollution.

Consequently, there will be new, improved, and more tailored cancer treatments that arise from the further research and development of plant extracts obtained via the ScCO_2_ approach. These advancements will not only expand possibilities in the battle against cancer and improve patient treatment processes, but also, the widespread adoption of this eco-friendly technology will have a substantial influence in combating environmental pollution and climate change.

## Figures and Tables

**Table 1:** Comparison of physical properties of gases, liquids, and SFs [10].

Solvent	Density (g/cm^3^)	Viscosity (gcm^−1^s^−1^)	Diffusion (cm^2^/s)
Gas ^a^	6 × 10^−4^–2 × 10^−3^	1 × 10^−5^–3 × 10^−5^	0.1–0.4
Liquid ^a^	0.6–1.6	2× 10^−4^–3× 10^−3^	2 × 10^−6^–2 × 10^−5^
SF ^b^	0.2–0.5	1 × 10^−4^–3 × 10^−4^	0.7 × 10^−3^
SF ^c^	0.4–0.9	3 × 10^−5^–9 × 10^−5^	2 × 10^−3^

^a^ P^0^ = 1 bar, T = 25 °C, ^b^ P = Pc, T = Tk, ^c^ P = 4Pc, T ≈ Tc.

**Table 2:** Critical values and properties of some SFs [13,14].

Fluid	Critical Temperature TC (°C)	Critical Pressure PC (atm)	Critical Density δC (g/cm^3^)	Density at 400 atm δ (g/cm^3^)	Boiling Temperature Tb (OC), (1 atm)
CO_2_	31.3	72.9	0.47	0.96	−73.5
N_2_O	36.5	71.7	0.45	0.94	-
NH_3_	132.5	112.5	0.24	0.40	−33.5
H_2_O	374.4	226.8	0.33	-	100
n-butane	152.0	37.5	0.23	0.50	−0.4
Ethane	32.4	48.3	0.20	-	−88.0
Ethanol	243.4	63.0	0.28	-	78.4
n-propane	96.8	42.0	0.22	-	−44.5
diethyl ether	193.6	36.3	0.28	-	34.6
trichloromethane	196.6	41.7	0.55	-	23.7
chlorotrifloromethane	28.8	39.0	0.58	-	−81.4

**Table 3:** Physical properties of ScCO_2_ at various temperatures and pressures [16].

Pressure (MPa)	Temperature (°C)	Density (Kg/m^3^)	Viscosity (Kg/m·s)
7.38	31	464	<2 × 10^−5^
24.5	39.85	879.5	8.2 × 10^−5^
	54.85	806.2	6.9 × 10^−5^
	69.85	730	5.7 × 10^−5^
17.6	39.85	814.6	7 × 10^−5^
	54.85	712.5	5.4 × 10^−5^
	69.85	599.1	4.1 × 10^−5^
10.8	39.85	676.1	4.7 × 10^−5^
	54.85	379.4	2.7 × 10^−5^
	69.85	283.6	2.4 × 10^−5^

**Table 4:** CO_2_ static dielectric constant [18].

T/°C	P/MPa	ɛ
−0.15	3.483	1.57785
9.85	4.493	1.52996
19.85	5.712	1.46753
24.85	6.433	1.46899
29.85	7.179	1.34809
31	7.377	<1.05

Saturation vapor pressures; data at higher pressures up 300 MPa and higher temperatures up to 79.85 °C are given in the original source.

**Table 6:** Anticancer activities of ScCO_2_ extracts of herbal sources.

Plants or Herbal Sources	Plant Material	Bioactive Compounds or Compound Classes	Extraction Conditions	Cancer Cell Lines	References
*Carica papaya*	Leaf juice	Vitamins and phytosterols	250 bar, 35 °C 180 min, 5 g material	SCC25 and HaCaT	[127]
Brazilian green propolis		Artepillin C	4000 psi 50 °C	HCT116	[128]
Vietnam *Ganoderma lucidum*	Extract	Triterpenoids, polysaccharides, phenolics, steroids, and alkaloids	37.5 MPa, 48 °C 1.5 h	KB, HepG2, Lu, and MCF7	[129]
*Cannabis*	Flower	-	30 min preheating at 140 °C, 40 MPa, and 50 °C	Caco-2, PC3, Hela, SiHa, and C33, HaCat, Vero, and L929	[130]
*Mangifera indica* L.	Seed kernel	Xanthones and gallic acid derivatives	50% water *v*/*v* in the feed extract and a feed/ScCO_2_ ratio of 0.0625 at 15 MPa	HT-29	[131]
*Baccharis uncinella*	Extract	-	150 and 200 bar and 60 °C	T24, U87, and Vero	[132]
*Xinjiang jujube*	Leaf	-	52.52 °C, 27.12 MPa, 113.42 min 0.44 mL/min co-solvent flow rate	A549	[34]
*Croton crassifolius* roots	Essential oil	-	25 MPa at 35 °C 30 min flow rate of 2 L CO_2_/min	A549, HeLa, HepG2, T24, MGC803, and HL-7702	[133]
*Helichrysum italicum*	Extract	Arzanol	100, 200, and 300 bars, 40 °C, 4 h and carbon dioxide flow 1.94 kg/h	MCF-7, HeLa, and MRC 5	[134]
*Zingiber officinale ROSCOE*	Rhizome	Terpene hydrocarbons, oxygenated terpenes, and other non-volatile compounds	35 MPa, 35 °C, a CO_2_ flow rate of 15 L/s, and 2 h	MDA-MB-231, A549, and HepG2	[135]
*Pistacia lentiscus* L.	Leaf	Germacrene D delta-cadinene alpha-pinene	-	Primary human endothelial cells	[136]
*Arnica montana* L.	Flower	-	60 °C, 30 MPa	WM266-4	[137]
*Azadirachta indica*	Leaf	Azadiractoids	-	HCT116 and HT29	[138]
